# Supplementary material for: Thermal dependence of the hydrated proton and optimal proton transfer in the protonated water hexamer
Source: Nat Commun. 2023 Oct 30;14:6930. doi: 10.1038/s41467-023-42366-4 (PMC10616126; doi:10.1038/s41467-023-42366-4)
Supplement: Supplementary file 1 — Supplementary Information [file 41467_2023_42366_MOESM1_ESM.pdf]

# Supplementary Information for "Thermal dependence of the hydrated proton and optimal proton transfer in the protonated water hexamer"

Félix Mouhat,[1] Matteo Peria,[2] Tommaso Morresi,[3] Rodolphe Vuilleumier,[4] Antonino Marco Saitta,[2] and Michele Casula[2,*]

[1]*Saint-Gobain Research Paris, 39 Quai Lucien Lefranc, 93300 Aubervilliers, France*

[2]*IMPMC, Sorbonne Université, CNRS, MNHN, UMR 7590, 4 Place Jussieu, 75252 Paris, France*

[3]*ECT*-Fondazione Bruno Kessler*, 286 Strada delle Tabarelle, 38123, Trento, Italy*

[4]*École normale supérieure, PSL Research University, Sorbonne Université, CNRS, Département de Chimie, PASTEUR, 24 Rue Lhomond, 75005 Paris, France*

(Dated: October 28, 2023)

* Correspondence and request for materials should be addressed to M.C. at michele.casula@sorbonne-universite.fr

## CONTENTS

Supplementary Note I. Zero-temperature electronic structure calculations 3
Supplementary Note I.1. Preparation and optimisation of quantum Monte Carlo (QMC) wave function 3
Supplementary Note I.2. Comparison between QMC and other electronic structure methods 4

Supplementary Note II. Finite-temperature calculations: QMC-driven classical and quantum Langevin dynamics 7
Supplementary Note II.1. Water dimer and Zundel ion as benchmark systems 8
Supplementary Note II.2. Protonated water hexamer 10

Supplementary Note III. Structural properties of protonated water clusters 11
Supplementary Note III.1. Role of solvation: Zundel ion versus protonated water hexamer 11
Supplementary Note III.2. $H_{13}O_6^+$ bidimensional distribution functions 12

Supplementary Note IV. Towards an accurate modeling of the potential energy surface (PES) 15

Supplementary Note V. Projected two-dimensional PES 18

Supplementary Note VI. Species population analysis 24

Supplementary Note VII. Proton transfer: adiabatic events versus quantum tunneling 26

Supplementary References 28

## Supplementary Note I. ZERO-TEMPERATURE ELECTRONIC STRUCTURE CALCULATIONS

### Supplementary Note I.1. Preparation and optimisation of quantum Monte Carlo (QMC) wave function

As described in the Methods Section, before running finite-temperature calculations, we optimize a QMC variational wave function $|\Psi_{\mathbf{q}}\rangle$ at zero temperature, written as a Jastrow Antisymmetrised Geminal Power (JAGP) ansatz[1]. During the finite-temperature molecular dynamics, energy and forces are computed at the variational Monte Carlo (VMC) level, based on the variational optimisation of the QMC wave function. Both Jastrow and AGP expansions are developed over a primitive O(3s2p1d) H(2s1p) and O(5s5p2d) H(4s2p) Gaussian basis functions, respectively. The primitive basis sets are then contracted using the geminal embedded orbitals (GEOs) scheme[2].

Previous works on the Zundel ion[3, 4] found that the optimal balance between accuracy and computational cost for the determinantal part is reached by the O[8]H[2] contracted GEO basis, in self-explaining notations. As the protonated water hexamer is a very similar system, in this work we used the same O[8]H[2] GEO contraction for the AGP part. Moreover, we further simplified the variational wave function previously developed for the Zundel ion, by contracting also the Jastrow basis set, using the same GEO embedding scheme. We tried different contraction sets, and tested them on the water dimer dissociation energy curve, as reported in Supplementary Fig. **1**. The water dimer is a stringent benchmark for the quality of our wave function, as it has a chemical complexity similar to the Zundel ion, with the main difference of being charge neutral. Charge neutrality allows us to directly probe the Jastrow capability of controlling charge fluctuations in the system, a fundamental property when coupled with the AGP determinantal part[5].

As shown in Supplementary Fig. **1**, we find a systematic improvement as the number of GEOs orbitals increases, with the O[6]H[2] set yielding energies very close to the Jastrow primitive basis set reference at all oxygen-oxygen distances. As reported in Supplementary Tab. **1**, this is obtained with a number $p$ of variational parameters significantly smaller than the one of the primitive basis set expansion. Thus, we used the O[6]H[2] GEO basis set for the Jastrow factor, and the O[8]H[2] GEO basis for the AGP part in all our subsequent

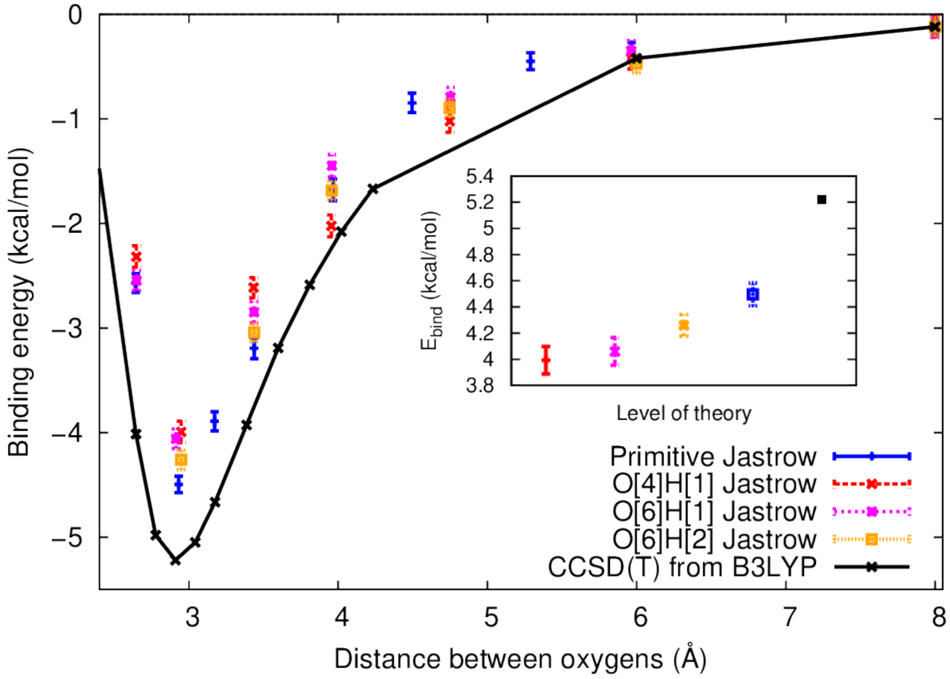


**Supplementary Figure 1**. Water dimer dissociation energy curve as a function of the oxygen-oxygen distance obtained by VMC, using different contracted basis sets in the Jastrow factor of a Jastrow-Slater wave function. Each trial wave function is built using the same basis set for the determinantal part, which is optimised together with the various Jastrow factors tested here. The black curve indicates the reference CCSD(T) result.

molecular dynamics (MD) simulations of the protonated water hexamer. This results into a total number of 6418 variational parameters, comprising $g^{a,b}_{\mu,\nu}$, $\lambda^{a,b}_{\mu,\nu}$, the parameters of the homogeneous one-body and two-body Jastrow factors, and the linear coefficients of the Jastrow and determinantal basis sets (see Methods Section for a detailed description of the wave function parameters).

A more extended description of the variational wave function can be found in Ref. [3].

### Supplementary Note I.2. Comparison between QMC and other electronic structure methods

To probe the microscopic rearrangement which triggers proton transfer (PT) processes in the protonated water hexamer at finite temperature, it is necessary to determine the potential energy surface (PES), in particular around the equilibrium geometry of the cluster.

| Basis set | $p$ | $E_{bind}$ (kcal/mol) |
| --- | --- | --- |
| Primitive Jastrow and primitive determinant | 6303 | 4.46(8) |
| Primitive Jastrow and O[8]H[2] GEO determinant | 2089 | 4.40(8) |
| O[6]H[2] GEO Jastrow and O[8]H[2] GEO determinant | 1283 | 4.26(8) |

**Supplementary Table 1**. Water dimer binding energies for QMC variational wave functions obtained with different types of basis set contractions. The corresponding number $p$ of variational parameters is also reported.

At variance with the Zundel cation, the protonated hexamer minimum energy configuration is asymmetric, implying that the hydrated proton is not equally shared between the two central water molecules.

In Supplementary Fig. **2**, we report the equilibrium position of the hydrated proton $H^+$ - expressed as distance $d_{H^+O_1}$ ($d_{H^+O_2}$) from the flanking oxygen atom $O_1$ ($O_2$) - as a function of the distance $d_{O_1O_2}$ between the two central oxygen atoms. Supplementary Fig. **2** shows the equilibrium geometries at constrained $d_{O_1O_2}$ obtained by various methods: density functional theory (DFT) with the PBE functional (dark green), DFT modified to include dispersive van der Waals (vdW) interactions in the DF2 implementation[6] (magenta), variational Monte Carlo (blue circles) and Møller-Plesset (MP2) (red triangles). The $d_{O_1O_2}$ distance corresponding to the global minimum is indicated by a vertical dashed line for each method.

The PBE functional predicts a short-Zundel-like symmetric global minimum, which is erroneous. More generally, this functional gives a poor description of the proton location when one stretches the $d_{O_1O_2}$ distance. After inclusion of dispersion effects in the DF2 functional, the geometric properties of the system are significantly improved, displaying a better agreement with both QMC and MP2. Nevertheless, the predicted equilibrium $d_{O_1O_2}$ is too large, which leads to an overly asymmetric cluster. Therefore, we expect the DF2 static barriers to be inaccurate, which is problematic in the perspective of studying PT at finite temperature by this method. Instead, MP2 and QMC are in excellent agreement, especially around $d_{O_1O_2} = 2.4$ Å.

In Supplementary Tab. **2** we report the equilibrium geometries yielded by PBE, DF2, MP2 and VMC, by showing the relevant distances involving the Zundel core of the protonated water hexamer. The VMC minimum geometry is in a very good agreement with the MP2

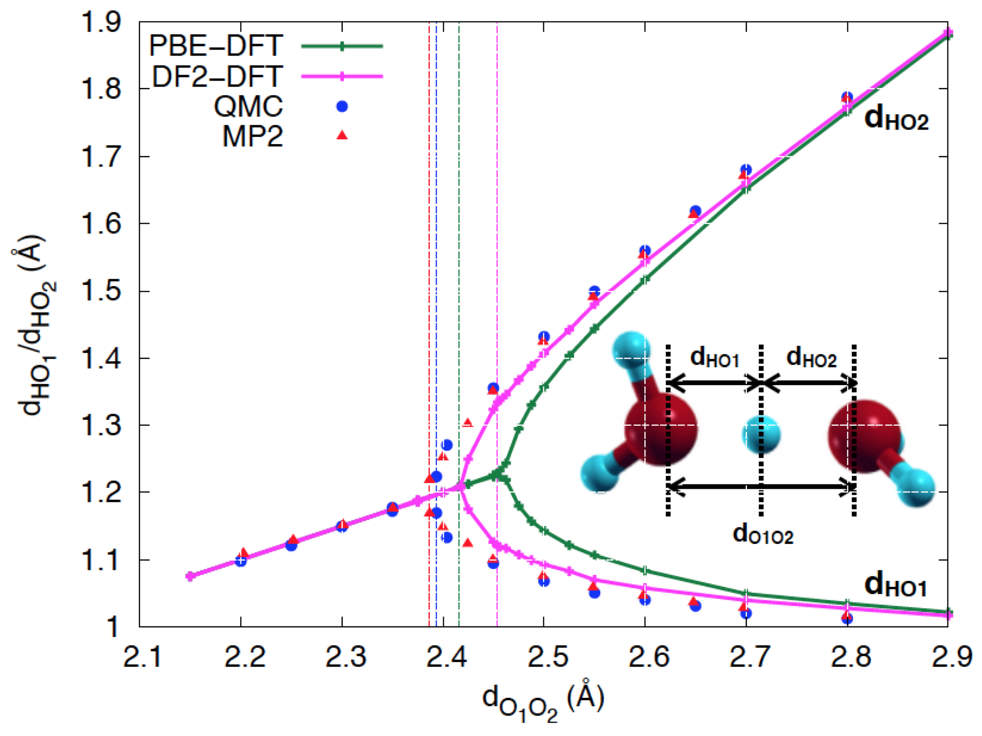


**Supplementary Figure 2**. Equilibrium position of the excess proton H - expressed as distance $d_{HO_1}$ ($d_{HO_2}$) from the flanking oxygen atom $O_1$ ($O_2$) - as a function of the separation $d_{O_1O_2}$ between the two central oxygen atoms, reported for different computational methods. Vertical dashed lines indicate the equilibrium $d_{O_1O_2}$ for each method.

one, with an accurate description of the excess proton localisation, as previously seen in Supplementary Fig. **2**. The largest discrepancy between VMC and MP2 is for the $\overline{OH}$ intramolecular distances, which are slightly shorter in VMC. However, the overall evolution of equilibrium position of the hydrated proton $H^+$ as a function of the oxygen-oxygen distance predicted by QMC follows well the one obtained by MP2, as one can see from Supplementary Fig. **2**.

Due to the central distorted H-bond, the hydrated proton motion from one flanking water molecule to another is conditioned by the necessary energy it should acquire to go across the PT static barrier. This quantity is defined as the energy difference between the equilibrium structure (asymmetric) and the symmetrised one, where the proton is located at the mid-point of the considered $d_{O_1O_2}$ distance. PT static barriers have been estimated at $d_{O_1O_2} = 2.45$ Å and 2.5 Å. The barriers, converted into effective temperatures, are reported

| Theory | $\overline{O_1O_2}$ | $\overline{O_1H^+}$ | $\overline{H^+O_2}$ | $\overline{O_1H_1}$ | $\overline{O_1H_2}$ | $\overline{O_2H_3}$ | $\overline{O_2H_4}$ |
|---|---|---|---|---|---|---|---|
| DFT-PBE | 2.4156 | 1.2078 | 1.2078 | 0.9935 | 0.9935 | 0.9935 | 0.9935 |
| DFT-DF2 | 2.4541 | 1.1196 | 1.3346 | 0.9913 | 0.9911 | 0.9797 | 0.9797 |
| MP2 | 2.3867 | 1.1690 | 1.2188 | 0.9877 | 0.9878 | 0.9847 | 0.9848 |
| VMC | 2.3930(5) | 1.1555(5) | 1.2375(5) | 0.9800(8) | 0.9798(8) | 0.9752(8) | 0.9748(8) |

**Supplementary Table 2**. Geometric properties (distances in Å) of the core of the protonated water hexamer minimum. Comparison between different computational methods.

in Supplementary Tab. **3** for different levels of theory.

| $d_{O_1O_2}$ (Å) | 2.45 Å | 2.50 Å |
|---|---|---|
| DFT-DF2 | 39 | 483 |
| CCSD(T) | 141 | 431 |
| MP2 | 85 | 327 |
| VMC | $195 \pm 25$ | $562 \pm 27$ |

**Supplementary Table 3**. Static symmetrisation barriers (in Kelvin) of the $H_{13}O_6^+$ cation at different $d_{O_1O_2}$ distances for various computational methods.

DFT-DF2 seems unable to predict accurate PT barriers due to the misplacement of the global energy minimum. This further motivates the use of correlated methods to describe the electronic PES, such as coupled cluster CCSD(T) and MP2 theories. Their values are closer to the barriers predicted by VMC. They differ between each other by 0.1-0.2 kcal/mol, a difference within chemical accuracy. However, the QMC method exhibits a milder scaling with the system size. Therefore, the QMC approach is certainly the best candidate to perform fully *ab initio* MD simulations of small protonated water clusters at an affordable computational cost.

## Supplementary Note II. FINITE-TEMPERATURE CALCULATIONS: QMC-DRIVEN CLASSICAL AND QUANTUM LANGEVIN DYNAMICS

We simulated the protonated water hexamer by treating the electrons at the VMC level (JAGP wave function) and the nuclei considered as quantum particles as well, within a path

integral (PI) formalism. To understand the impact of quantum effects, simulations with classical nuclei have also been performed. The Langevin dynamics (LD) algorithms used have been described in the Methods Section.

### Supplementary Note II.1. Water dimer and Zundel ion as benchmark systems

To check the accuracy of our QMC variational ansatz in describing the H-bond and the $O_1$-$H^+$-$O_2$ bond, we carried out QMC-driven PIMD simulations of the water dimer and the Zundel complex, and compared them against the results obtained with CCSD(T)-derived PESs[7, 8], available for these systems.

In Supplementary Fig. **3**, we plot the radial distribution function $g(r)$ computed in the water dimer at 200 K for the *intra*-molecular oxygen-hydrogen atoms (Supplementary Fig. **3**, left panel) and for the *inter*-molecular oxygen-oxygen atomic pairs (Supplementary Fig. **3**, right panel). These distribution functions have been obtained from PIMD simulations based on the newly generated CCSD(T)-precision q-AQUA potential[7], and based on our QMC variational ansatz. Here, our variational Monte Carlo wavefunction is made of a Jastrow correlated Slater determinant, developed on the contracted basis set introduced in Supplementary Note.I.1, namely the O[6]H[2] GEO basis for the Jastrow factor and the O[8]H[2] GEO basis for the determinantal part. In this particular case, the time evolution of the PIMD simulations has been taken as long as $\approx 70$ ps, with a time step of 1 fs, in order to have a good statistics on the oxygen-oxygen $g(r)$, which is particularly noisy to compute in the water dimer.

In Supplementary Fig. **4**, we plot the $O_1$-$O_2$ pair correlation function of the Zundel ion at 300 K, obtained by PESs computed at different levels of theory for the electronic part: CCCD(T)[8], a QMC-JAGP ansatz with a Jastrow factor expanded in a primitive basis (already used in the PIMD calculations of the Zundel ion carried out in Ref. [4]), and a QMC-JAGP ansatz with a Jastrow factor contracted into a O[6]H[2] GEO basis set, introduced in Supplementary Note.I.1, and used in this system for the first time here.

As one can see from Supplementary Figs. **3** and **4**, the pair correlation functions yielded by the QMC-PES are in a good statistical agreement with the ones generated by PIMD simulations driven by CCSD(T) PESs, yielding similar peaks position and width. This shows the quality of the QMC PES in a region around the minimum relevant for the quantum-

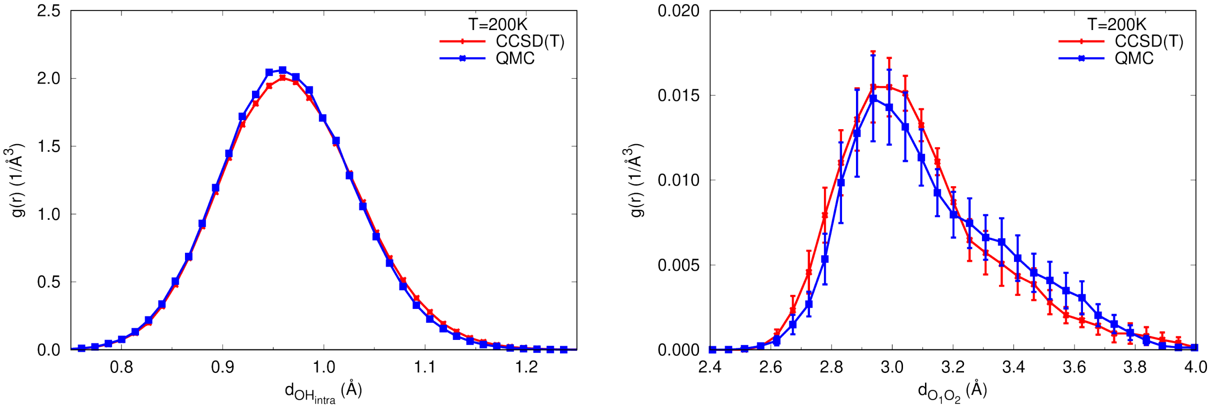


**Supplementary Figure 3**. Comparison between PIMD simulations of the water dimer driven by the q-AQUA potential (CCSD(T)) and our variational Jastrow-Slater wavefunction (QMC). Left panel: Radial distribution function $g(r)$ obtained at 200 K for the *intra*-molecular oxygen-hydrogen pairs summed up. Right panel: oxygen-oxygen $g(r)$ at the same temperature. In these simulations, we used 32 beads in both CCSD(T) and QMC cases.

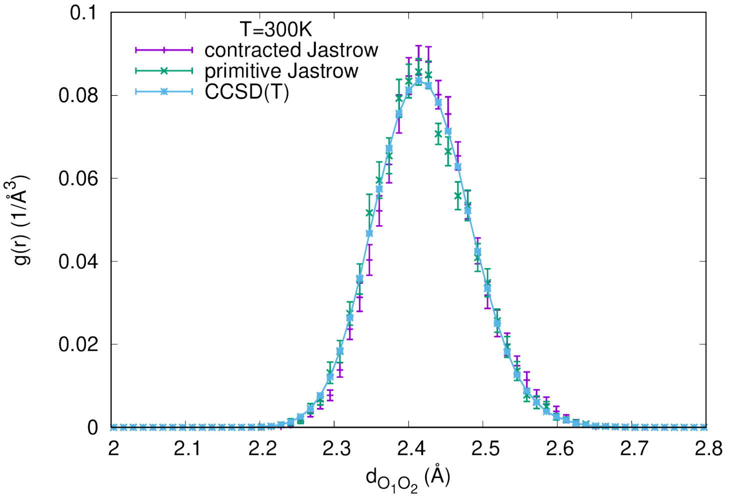


**Supplementary Figure 4**. $O_1$-$O_2$ pair correlation function computed for the Zundel complex by means of different approaches: the QMC-driven PIMD using a wave function with a primitive[4] and an O[6]H[2] GEO contracted Jastrow factor. The results are compared with a PIMD simulations based on CCSD(T) forces[8].

thermal motion of these aqueous systems.

**Supplementary Note II.2. Protonated water hexamer**

In Supplementary Tab. **4**, we report the complete list of VMC+PILD simulations done for the protonated water hexamer. Owing to their importance, particularly long simulations are performed for the quantum case at temperatures of 50, 100, 200, and 300 K. In all simulations, we generated at least 1850000 electronic Monte Carlo configurations to optimise the wave function at each step of MD or PIMD dynamics. The resulting CPU time per time step is reported in the Table. Notice that in our framework PIMD is not more costly than classical MD, thanks to the "bead grouping approximation".

**Supplementary Table 4**. Summary of the simulations carried out in this work. In both classical and quantum calculations, a time step $\delta t$ of 1 ft is used for all temperatures. The CPU time per time step ($t_{\mathrm{iteration}}$) is also reported in hours. $^1$: calculations done on 68-core Intel Xeon Phi 7250 CPU (Knights Landing) nodes at 1.40 GHz. $^2$: calculations done on dual-processor (2x64 cores) AMD Rome (Epyc) compute nodes at 2.6 GHz.

| | quantum simulations | | | classical simulations | |
|---|---|---|---|---|---|
| $T$(K) | $N_{\mathrm{beads}}$ | $N_{\mathrm{iterations}}$ | $t_{\mathrm{iteration}}$(h) | $N_{\mathrm{iterations}}$ | $t_{\mathrm{iteration}}$(h) |
| 50 | 128 | 35282 | $119.4^1$ | - | - |
| 100 | 128 | 52184 | $24.4^2$ | 21454 | $42.0^2$ |
| 150 | 64 | 11218 | - | - | - |
| 200 | 64 | 32553 | $95.7^1$ | 20478 | $103.6^1$ |
| 250 | 32 | 23912 | $92.2^1$ | 24154 | $123.5^1$ |
| 300 | 32 | 31929 | $106.3^1$ | 22656 | $109.9^1$ |
| 350 | 32 | 18489 | $102.4^1$ | 26481 | $130.5^1$ |
| 400 | 32 | 23026 | $120.9^1$ | 27517 | $134.0^1$ |

## Supplementary Note III. STRUCTURAL PROPERTIES OF PROTONATED WATER CLUSTERS

### Supplementary Note III.1. Role of solvation: Zundel ion versus protonated water hexamer

As mentioned in the main text of the paper, the protonated water hexamer is the smallest water cluster including the full first-solvation shell, due to the presence of a Zundel core surrounded by 4 solvating $H_2O$ molecules. To quantify the impact of the solvation shell on the Zundel core, we compare in Supplementary Fig. **5** the $O_1$-$O_2$ potential, $V_{O_1O_2}$ (left), and the corresponding classical equilibrium geometry (right) of the two clusters at various $d_{O_1O_2}$ (distance between the 2 central oxygen atoms). At short $d_{O_1O_2}$, the slope of the protonated hexamer $V_{O_1O_2}$ is slightly larger than the Zundel one, due to a greater electrostatic repulsion because of steric hindrance. At large $d_{O_1O_2}$, the protonated hexamer PES is softer than the Zundel one, because the solvating $H_2O$ molecules enhance the polarisability of the core atoms. As explained in the paper, the balance between short- and long-range repulsion, once supplemented with the zero-point energie (ZPE), is key to quantify the relative abundance of short-Zundel and distorted-Eigen configurations, and thus, it allows for a quantitative understanding of the PT mechanism.

We also find the $H_{13}O_6^+$ equilibrium $d_{O_1O_2}$, represented by a vertical dashed line in Supplementary Fig. **5**, to be $\sim 0.01$ Å larger than the $H_5O_2^+$ one. More importantly, at variance with the Zundel cation which is centrosymmetric, the protonated water hexamer equilibrium geometry is *asymmetric* with classical ions. This fundamental symmetry modification of the PES is induced by solvation effects, which tend to stabilize the hexamer into its elongated-Zundel configuration. This can rationalise some THz/FTIR absorption spectroscopy fingerprints of the solvated proton[10], which have been related to a fast inter-conversion between the (distorted-)Eigen and (short-)Zundel forms.

Furthermore, it is noteworthy that at fixed $d_{O_1O_2}$, the predicted barriers are larger in $H_{13}O_6^+$ (Supplementary Tab. **3**) than in the Zundel cation[3]. This is clearly due to the additional price that must be paid for the rearrangement of the molecules in the solvation shell during the PT process.

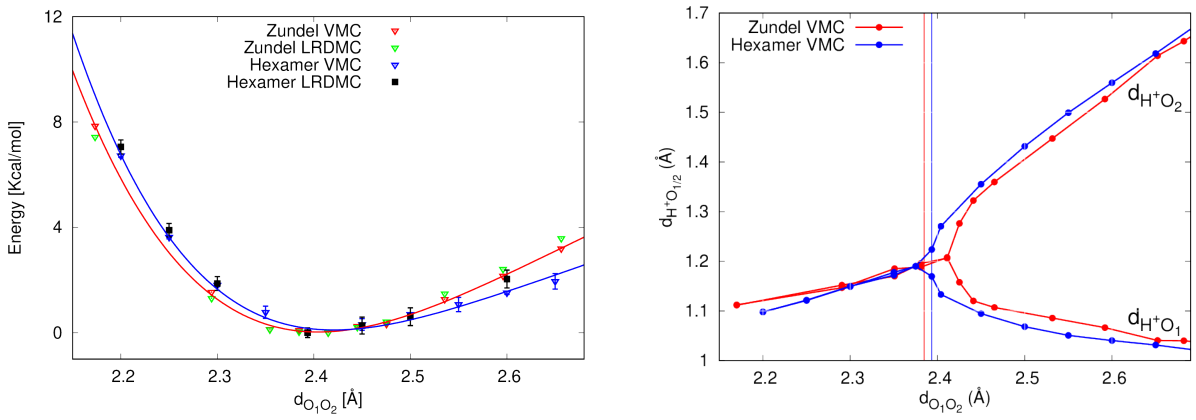


**Supplementary Figure 5**. Comparison of the protonated water dimer and hexamer $V_{O_1O_2}$ potential (left) and equilibrium geometry (right) as a function of the (central) oxygen-oxygen distance $d_{O_1O_2}$. Vertical dashed lines indicate the corresponding equilibrium $d_{O_1O_2}$. Notice that VMC and lattice regularised diffusion Monte Carlo (LRDMC)[9] energies are in nice statistical agreement for $d_{O_1O_2} \in [2.3, 2.6]$ Å, the phase-space range explored by our MD simulations.

### Supplementary Note III.2. $H_{13}O_6^+$ bidimensional distribution functions

We report some finite-temperature properties of the $H_{13}O_6^+$ system, studied by looking at the bidimensional distributions functions $\rho_{2D}$, which correlate the distance between the central proton and the neighbouring oxygen atoms ($d_{H^+O_1}$, $d_{H^+O_2}$) with $d_{O_1O_2}$. They are shown in Supplementary Figs. **6** and **7**, for quantum and classical simulations, respectively.

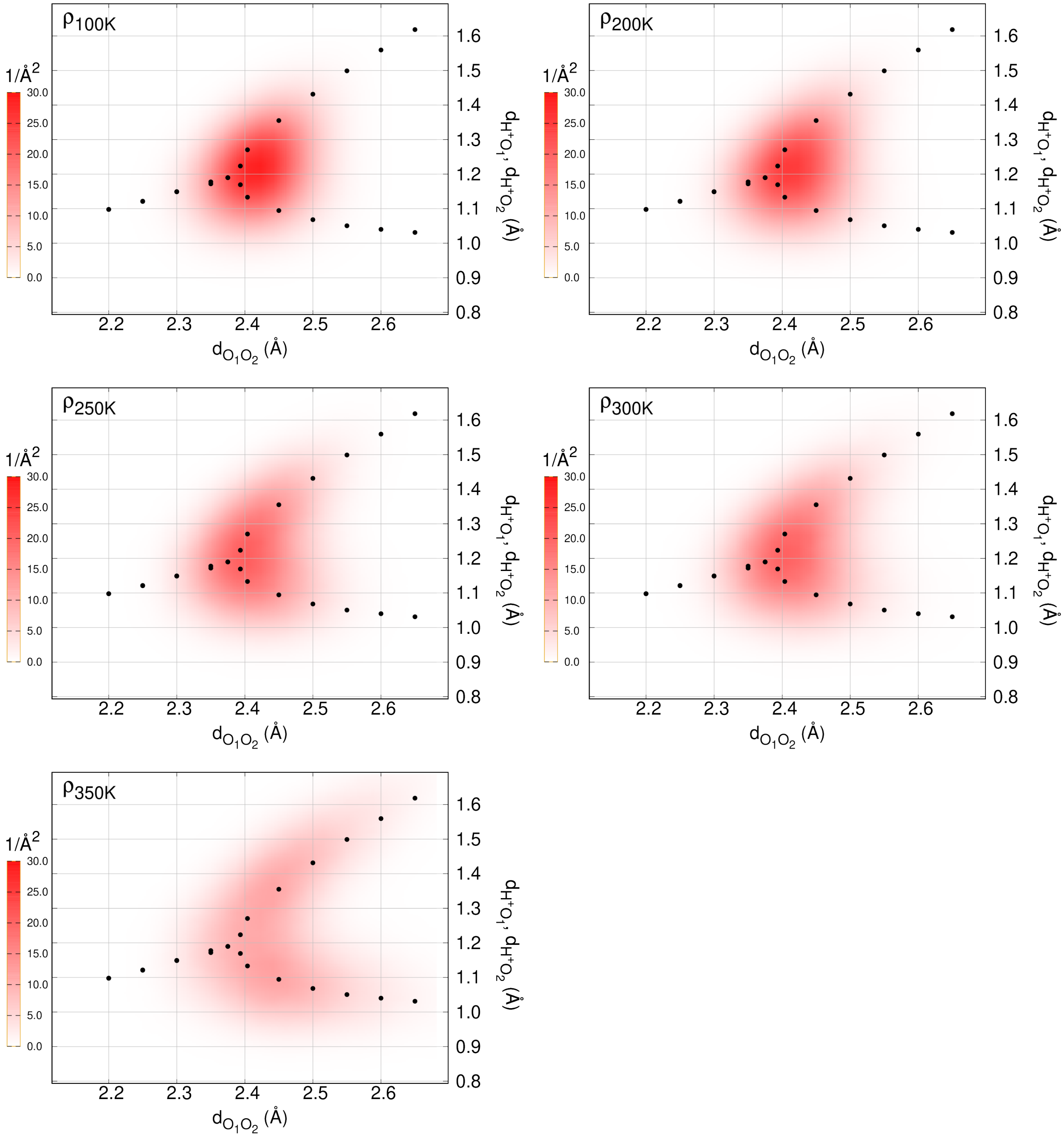


**Supplementary Figure 6**. $\rho_{2D}$ computed from VMC-PIMD simulations at different temperatures. Source data are provided as a Source Data file.

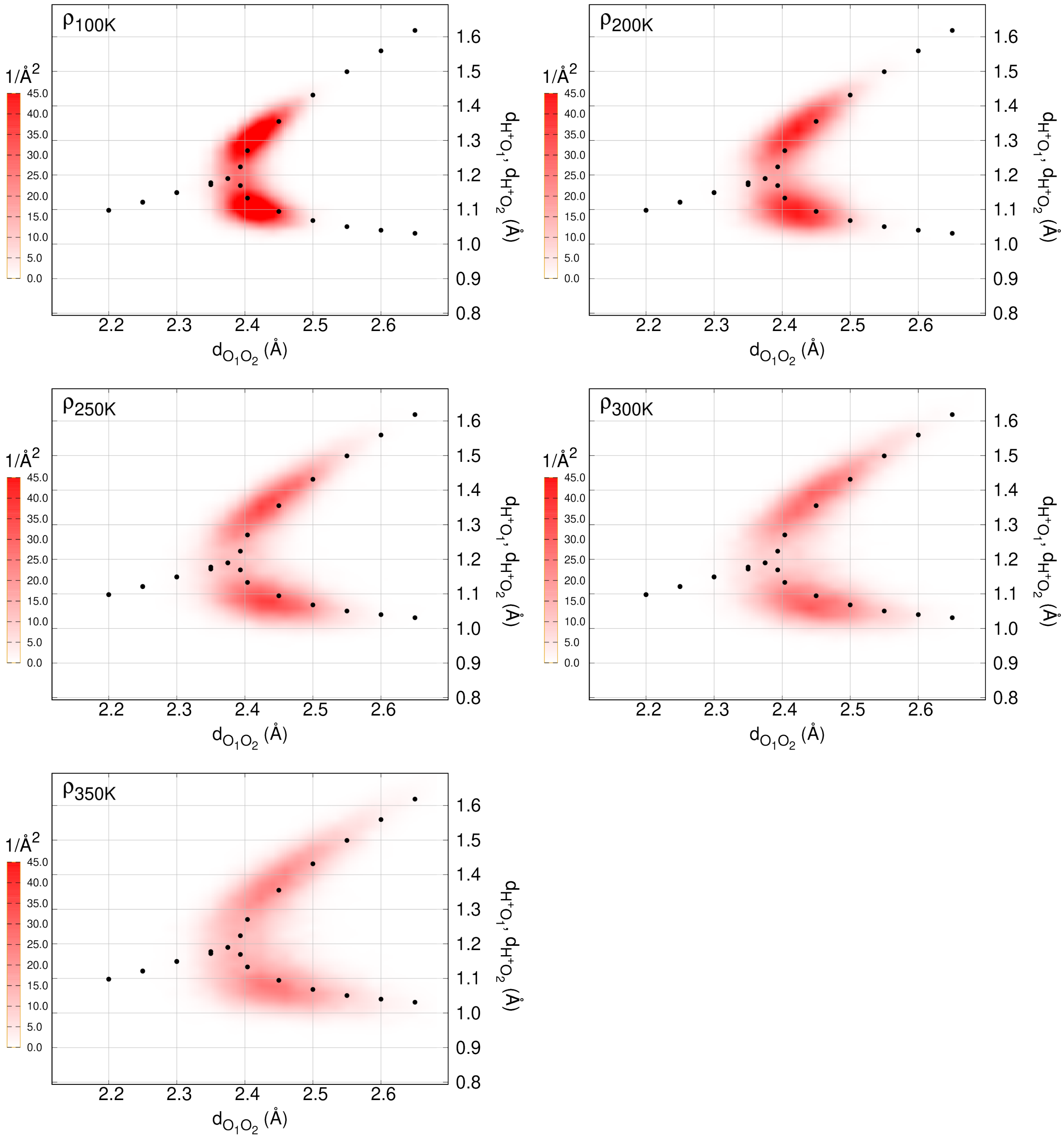


**Supplementary Figure 7**. $\rho_{2D}$ computed from VMC-MD simulations, with classical nuclei, at different temperatures. Source data are provided as a Source Data file.

The difference between quantum and classical distributions is striking. The impact of nuclear quantum effects (NQEs) on the temperature-dependence of $H_{13}O_6^+$ structural properties and PT mechanism are analysed and detailed in the main text, based on the proton density distributions shown in Supplementary Figs. **6** and **7**.

## Supplementary Note IV. TOWARDS AN ACCURATE MODELING OF THE POTENTIAL ENERGY SURFACE (PES)

We exploit the calculation of VMC forces not only to perform QMC-driven classical and quantum LD, but also to extract the best PES fitting functional form for the excess proton and for the water-water interaction in the Zundel core. The final goal is to derive the two-dimensional (2D) model potential $V_{2D} = V_{2D}(d_{O_1O_2}, \delta)$, where $d_{O_1O_2}$ is the distance between the two central oxygen atoms and $\delta$ is the proton sharing coordinate, referenced to the midpoint of the $O_1H^+O_2$ complex: $\delta \equiv \tilde{d}_{O_{1/2}H^+} - d_{O_1O_2}/2$, with $\tilde{d}_{O_{1/2}H^+}$ the $O_{1/2}$-$H^+$ distance projected onto the $O_1O_2$ direction. The projection of the full interatomic potential on the restricted 2D manifold is done by integrating the other degrees of freedom over the thermal partition function, sampled during the MD dynamics, i.e. $V_{2D}(d_{O_1O_2}, \delta) \equiv \langle V(q_1, q_2, \mathbf{q}_{N-2})\delta(q_1 - d_{O_1O_2})\delta(q_2 - \delta)\rangle$, where $\langle \ldots \rangle$ is the average over the partition function of the classical/quantum statistical ensemble at fixed temperature, and $V$ is the $3N$-dimensional potential depending on the generalised nuclear coordinates of the full system. Analogously, one can define the one-dimensional (1D) potential acting between $O_1$ and $O_2$ as $V_{1D} = V_{1D}(d_{O_1O_2}) \equiv \langle V(q_1, q_2, \mathbf{q}_{N-2})\delta(q_1 - d_{O_1O_2})\rangle$, according to previous notations. Derivatives of the previous potentials with respect to $d_{O_1O_2}$ and/or $\delta$ can be defined in the same way. For instance, $\partial V_{2D}/\partial\delta \equiv \langle \partial V(q_1, q_2, \mathbf{q}_{N-2})/\partial q_2 \; \delta(q_1 - d_{O_1O_2})\delta(q_2 - \delta)\rangle$, and $\partial V_{1D}/\partial d_{O_1O_2} \equiv \langle \partial V(q_1, q_2, \mathbf{q}_{N-2})/\partial q_1 \; \delta(q_1 - d_{O_1O_2})\rangle$.

Given these definitions, we can proceed with the calculations of the corresponding quantities with the aim at modeling the potentials $V_{1D}$ and $V_{2D}$. To do so, we will integrate the other degrees of freedom using the classical Boltzmann distribution in $\langle \ldots \rangle$, as generated by the QMC-driven classical Langevin dynamics at 100, 250 and 350 K. Employing the classical partition function has the advantage that the potentials *sampled* in this way will tend to the original PES of the system as $\beta \to \infty$, while the quantum partition function will lead to averaged potentials biased by quantum fluctuations even in the zero temperature limit. To compute these quantities from an MD sampling, the $\delta$-functions in their definitions above are replaced by bins, whose size is given by the spacing between neighbouring points.

In Supplementary Fig. **8**, we study the $V_{1D}(d_{O_1O_2})$ potential depending on the water-water distance $d_{O_1O_2}$ (left column), and its derivative $\partial V_{1D}/\partial d_{O_1O_2}$ (right column). As one can see, the energy profile, at the left-hand side, is much more noisy than the behavior of its

gradient, from where we can extract a precise value of the equilibrium $d_{O_1O_2}$ distance, and the evolution of the potential around the minimum. This shows the advantage of computing QMC forces in order to determine the PES, and suggests that a robust way of deriving the $V_{1D}$ potential is by fitting and integrating its derivatives, rather than by directly fitting the energies.

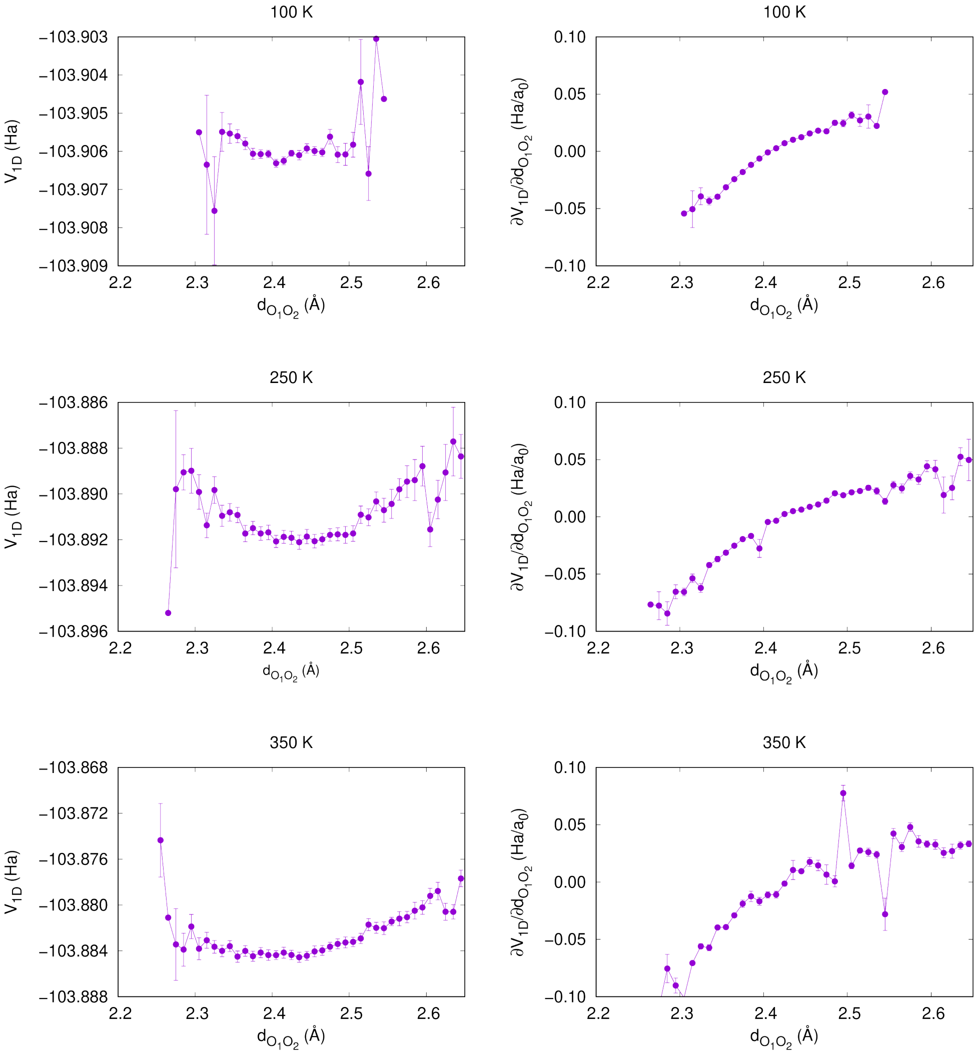


**Supplementary Figure 8**. Left-hand side: total energy variation of the cluster as a function of the $d_{O_1O_2}$ distance ($V_{1D}$). Right-hand side: sum of the energy gradients with respect to $\mathbf{q}_{O_1}$ and $\mathbf{q}_{O_2}$ variations projected along the $O_1O_2$ direction, for classical simulations at different temperatures. This corresponds to $\partial V_{1D}/\partial d_{O_1O_2}$, resulting in the force that drives the $O_1$-$O_2$ stretching mode. Source data are provided.

In Supplementary Fig. **9**, we study the $V_{2D}(d_{O_1O_2}, \delta)$ potential depending on the proton coordinate $\delta$, at various (fixed) $d_{O_1O_2}$ distances. In the left column, we show $\partial V_{2D}/\partial\delta$ in a

contour plot as a function of both $d_{O_1O_2}$ and $\delta$. Positive (negative) values of $\partial V_{2D}/\partial\delta$ are coloured in red (blue). The white region indicates the extrema of the 2D-PES. The classical proton is clearly asymmetric for $d_{O_1O_2} \gtrsim 2.37$ Å, with a minimum departing from the $\delta = 0$ axis. In the right column, the same information is provided by superposing $\partial V_{2D}/\partial\delta$ plotted as a function of $\delta$ and taken at fixed $d_{O_1O_2}$ distances.

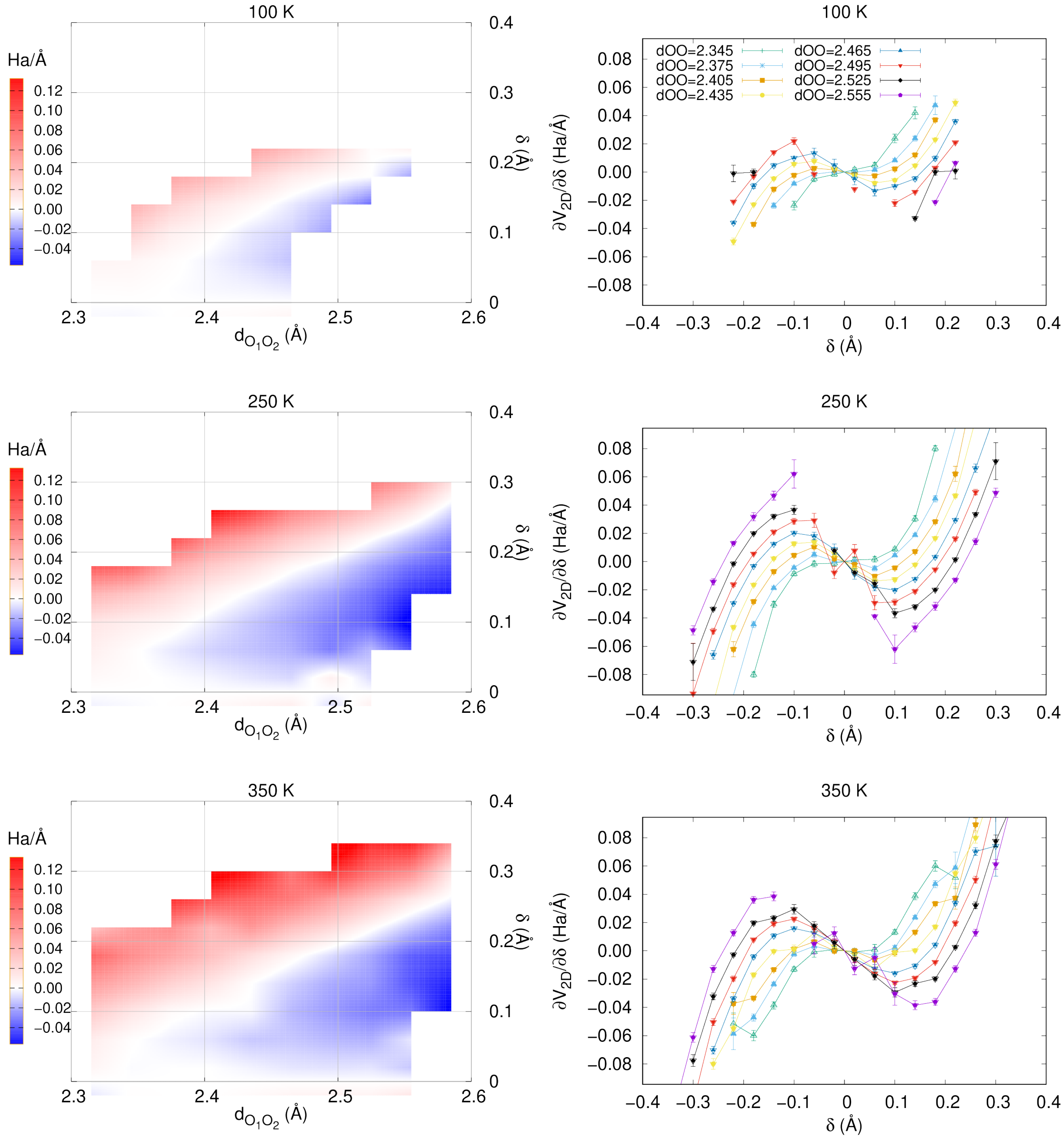


**Supplementary Figure 9**. Left column: contour plot of $\partial V_{2D}/\partial\delta$ as a function of both $d_{O_1O_2}$ and $\delta$. Right column: superposition of $\partial V_{2D}/\partial\delta$, plotted as a function of $\delta$ at various (fixed) $d_{O_1O_2}$ values. The force acting on $H^+$ projected along the $O_1O_2$ direction is given by $-\partial V_{2D}/\partial\delta$. Notice that the size of the $(d_{O_1O_2}, \delta)$ space accessible by MD to sample these quantities increases as a function of the temperature.

## Supplementary Note V. PROJECTED TWO-DIMENSIONAL PES

Using the data obtained in Supplementary Note IV, let us determine an analytic form for the $V_{2D}(d_{O_1O_2}, \delta)$ potential, which depends on both $d_{O_1O_2}$ and $\delta$ coordinates. This will take into account the variation of the proton-oxygen potential along the proton shuttling mode as the distance between the two inner water molecules varies.

We first derive the $V_{1D}$ potential between the two water molecules, which depends only on the $d_{O_1O_2}$ stretching coordinate, by fitting the derivatives shown in Supplementary Fig. **8**, for the simulation at 100 K, which yields less noisy datapoints than the one at higher temperatures. As fitting function, we choose the Morse potential, such that:

$$V_{1D}(x) = b_{\mathrm{Morse}} \left(\exp(-2c_{\mathrm{Morse}}(x - d_{\mathrm{Morse}})) - 2\exp(-c_{\mathrm{Morse}}(x - d_{\mathrm{Morse}}))\right) - b_{\mathrm{Morse}}, \qquad (1)$$

where we have chosen to set the zero of energy at the potential minimum. The results of the fit are plotted in Supplementary Fig. **10**, together with the potential derivatives evaluated by classical MD driven by QMC forces at 100 K, 250 K and 350 K.

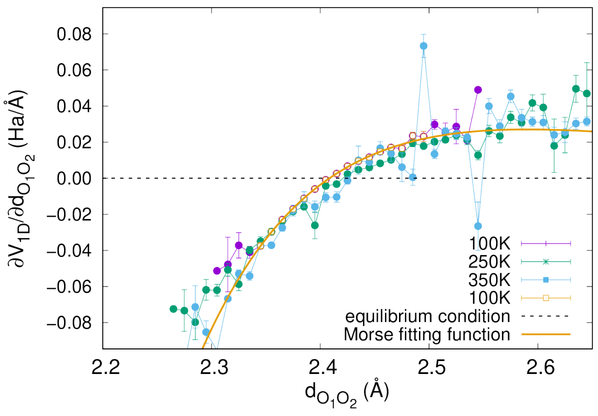


**Supplementary Figure 10**. Fit of the QMC estimates of $\partial V_{1D}/\partial \mathrm{d}_{O_1O_2}$ at 100 K. As fitting function we have used the derivative of the $V_{1D}$ Morse potential, as defined in Eq. 1. Source data are provided as a Source Data file.

From this analysis, the estimated equilibrium distance between two water molecules in the Zundel core of the protonated water hexamer is 2.408 Å at 100 K, in good agreement with the analysis based on the radial distribution function reported in Fig. 2 of the main text.

While the data derived from QMC-MD simulations are less noisy at 100 K, they however explore a smaller phase space, due to a probability density distribution more localised in the $(d_{\mathrm{O}_1\mathrm{O}_2}, \delta)$ space at lower temperatures. This turns out to be a problem, if one aims at estimating the behavior of the $V_{\mathrm{2D}}(d_{\mathrm{O}_1\mathrm{O}_2}, \delta)$ potential not only around its equilibrium geometry but also over its tails. A way to overcome this issue within the projection framework described in Supplementary Note IV, is to sample the projected potential from QMC-MD simulations carried out at higher temperatures. As clearly shown in Supplementary Fig. **9**, at 250 K and 350 K the $V_{\mathrm{2D}}$ behavior can be evaluated on a much larger window in both $\delta$ and $d_{\mathrm{O}_1\mathrm{O}_2}$ directions. Moreover, we can increase the statistics of higher temperatures datapoints by averaging the 250 K and 350 K estimates.

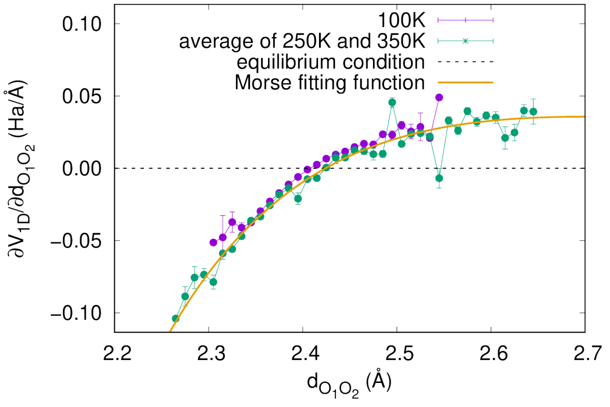


**Supplementary Figure 11**. Fit of the QMC forces using $\partial V_{\mathrm{1D}}/\partial x$ as fitting function, where the Morse potential $V_{\mathrm{1D}}(x)$ is defined in Eq. 1 and the fitted dataset is taken by averaging the outcome of 250 K and 350 K simulations. Source data are provided as a Source Data file.

We then fit the $V_{\mathrm{1D}}$ potential in Eq. 1 by using a dataset averaged over 250 K and 350 K. The corresponding Morse potential fit is reported in Supplementary Fig. **11**. From this analysis, the equilibrium distance between two water molecules in the Zundel core of the protonated water hexamer is 2.425Å at $\approx$ 300 K, again in a satisfactory agreement with the analysis based on the radial distribution function reported in Fig. 2 of the main text. Fitting over these datapoints leads to an increase of the equilibrium distance by 0.16Å as the temperature is raised from 100 K to $\approx$ 300 K. The average based on the radial distribution function of the full VMC-MD simulations yields a cluster expansion of $\approx$ 0.25Å.

The Morse potential determined from points computed at 100 K and the one from points

averaged over 250 K and 350 K are plotted in Supplementary Fig. **12**, where the two fitting functions are superimposed. The ZPE analysis described in the main text of the paper is carried out using the dataset averaged over 250 K and 350 K. As one can see in Supplementary Fig. **12**, in the energy range below 1000 K, the two curves are just shifted from one another, having nearly the same curvature around the minimum. Therefore, the conclusions on the ZPE effect reached by using a model potential projected at larger temperatures would not be different from the ones one could reach using the potential derived at 100 K.

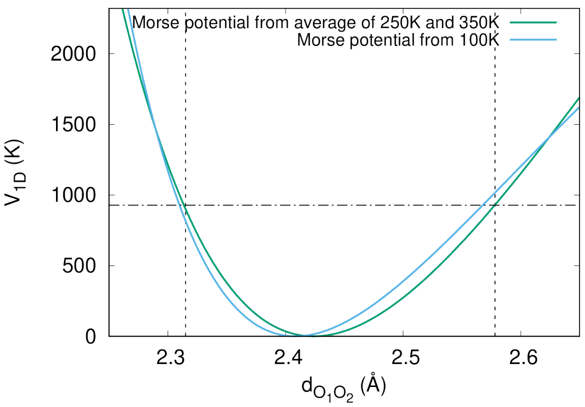


**Supplementary Figure 12**. $V_{1D}$ determined from classical MD at 100 K and from the averaged dataset of classical MD at 250 K and 350 K. The energy is expressed in Kelvin. The horizontal and vertical dashed lines are guides for the eye. Source data are provided as a Source Data file.

As a second step, we derive the $V_{2D}(d_{O_1O_2}, \delta)$ potential. For every $d_{O_1O_2}$ slice, in Supplementary Fig. **13** we plot the estimated values of the $\partial V_{2D}/\partial \delta$ derivative as a function of $\delta$, computed by averaging over the 250 K and 350 K classical MD samples, as we did for the $V_{1D}$ potential.

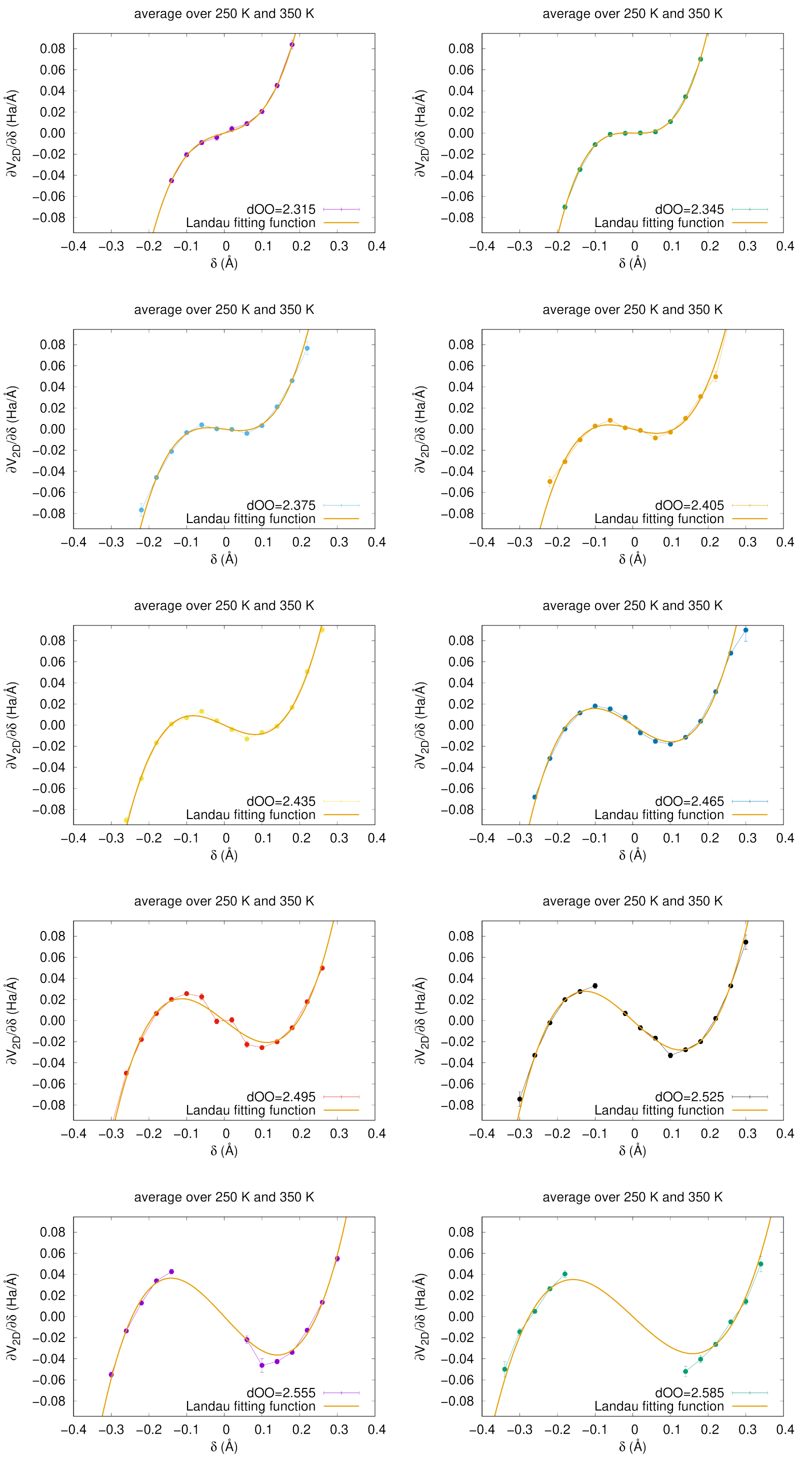


**Supplementary Figure 13**. Fit of the QMC forces using $\partial V_{d_{\mathrm{O_1O_2}}}(x)/\partial x$ as fitting function for different $d_{\mathrm{O_1O_2}}$, where the potential $V_{d_{\mathrm{O_1O_2}}}(x)$ is defined in Eq. 2. The points are calculated as average over the two temperatures of 250 K and 350 K. Source data are provided.

At every $d_{O_1O_2}$, we fit the derivative of the energy with respect to $\delta$, by using a symmetric quartic function, i.e. a Landau potential, as fitting model for the energy dependence:

$$V_{d_{O_1O_2}}(x) = a_{\text{Landau}} + b_{\text{Landau}} x^2 + c_{\text{Landau}} x^4 \qquad \text{at fixed } d_{O_1O_2} \text{ distance.} \tag{2}$$

The fits for selected $d_{O_1O_2}$ values are also reported in Supplementary Fig. **13**. The parameters $a_{\text{Landau}}$, $b_{\text{Landau}}$ and $c_{\text{Landau}}$ have thus an implicit dependence on $d_{O_1O_2}$, which need to be further included in the full $V_{2D}(d_{O_1O_2}, \delta)$ function . We found that a good parametrisation for $b_{\text{Landau}}$ is given by:

$$b_{\text{Landau}}(d_{O_1O_2}) = \alpha + \beta d_{O_1O_2}, \tag{3}$$

while for $c_{\text{Landau}}$ is:

$$c_{\text{Landau}}(d_{O_1O_2}) = \epsilon \exp(-\gamma d_{O_1O_2}). \tag{4}$$

Note that the $d_{O_1O_2}$-dependence in Eq. 4 guarantees that the potential in Eq. 2 always binds for $\epsilon > 0$. Supplementary Fig. **14** demonstrates how this dependence, shown by the parameters evolution, is well taken into account by the functional forms in Eqs. 3 and 4.

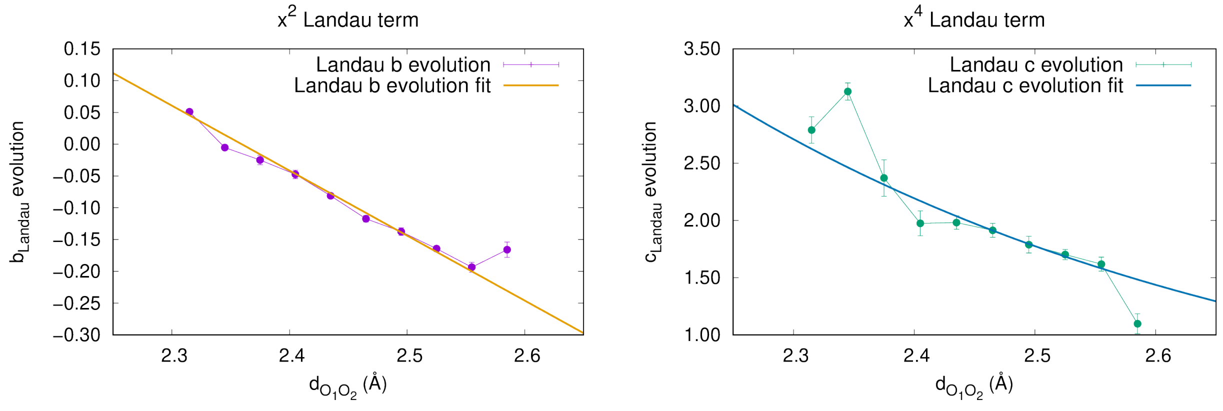


**Supplementary Figure 14**. Fit of the $b_{\text{Landau}}$ and $c_{\text{Landau}}$ dependence on the $d_{O_1O_2}$ distance, based on the functional forms in Eqs. 3 and 4. Source data are provided as a Source Data file.

From Eq. 3 and its fitting parameters, the bifurcation point turns out to be located at $d_{\text{symm}} \approx 2.37$Å, in quite good agreement with the relaxation of the ground state geometry.

The final 2D potential $V_{2D}(d_{O_1O_2}, \delta)$ is thus fully determined by the following function:

$$V_{2D}(d_{O_1O_2}, \delta) = a_{\text{Landau}}(d_{O_1O_2}) + b_{\text{Landau}}(d_{O_1O_2})\delta^2 + c_{\text{Landau}}(d_{O_1O_2})\delta^4, \tag{5}$$

with $b_{\text{Landau}}(d_{\text{O}_1\text{O}_2})$ and $c_{\text{Landau}}(d_{\text{O}_1\text{O}_2})$ already defined in Eqs. 3 and 4, respectively, while $a_{\text{Landau}}(d_{\text{O}_1\text{O}_2})$ is defined as follows:

$$a_{\text{Landau}}(d_{\text{O}_1\text{O}_2}) = V_{1\text{D}}(d_{\text{O}_1\text{O}_2}) + \Delta(d_{\text{O}_1\text{O}_2}). \tag{6}$$

In the above Equation, $\Delta(d_{\text{O}_1\text{O}_2})$ is the proton barrier of the Landau potential $V_{d_{\text{O}_1\text{O}_2}}(x)$ in Eq. 2, such that the bottom of $V_{2\text{D}}(d_{\text{O}_1\text{O}_2}, \delta)$ at a given $d_{\text{O}_1\text{O}_2}$ distance follows exactly the Morse potential $V_{1\text{D}}$ in Eq. 1. In particular, $\Delta(d_{\text{O}_1\text{O}_2})$ reads:

$$\Delta(d_{\text{O}_1\text{O}_2}) = \begin{cases} 0, & \text{if } d_{\text{O}_1\text{O}_2} \leq d_{\text{symm}} \\ \frac{b^2_{\text{Landau}}(d_{\text{O}_1\text{O}_2})}{4c_{\text{Landau}}(d_{\text{O}_1\text{O}_2})}, & \text{otherwise.} \end{cases} \tag{7}$$

The resulting 2D potential $V_{2\text{D}}(d_{\text{O}_1\text{O}_2}, \delta)$ and its derivative with respect to $\delta$ are drawn in the contour plot of Supplementary Fig. **15**. $\frac{\partial}{\partial\delta}V_{2\text{D}}(d_{\text{O}_1\text{O}_2}, \delta)$ compares very well with the

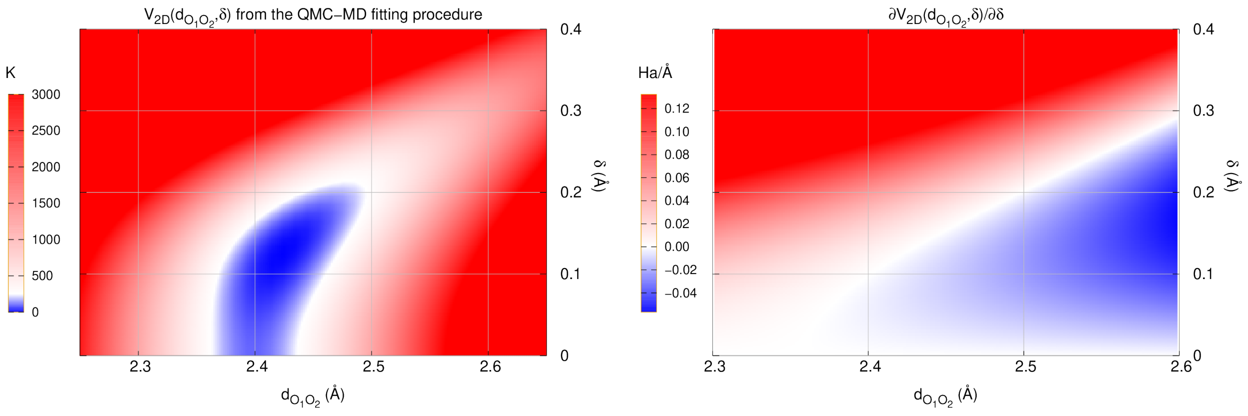


**Supplementary Figure 15**. Left panel: contour plot of the $V_{2\text{D}}(d_{\text{O}_1\text{O}_2}, \delta)$ 2D model potential. Right panel: contour plot of the model-potential derivative $\frac{\partial}{\partial\delta}V_{2\text{D}}(d_{\text{O}_1\text{O}_2}, \delta)$, to be compared with the contour plot of the mid- and bottom-left panels of Supplementary Fig. **9**, directly obtained from MD sampled datapoints.

same quantity directly evaluated by QMC-driven classical MD at both 250 K and 350 K, as shown in Supplementary Fig. **9**, mid- and bottom-left panels. This is an *a posteriori* check of the quality of our 2D-PES determination.

As we have seen, there is a residual temperature dependence in the determination of $V_{2\text{D}}(d_{\text{O}_1\text{O}_2}, \delta)$, due to the projection scheme employed. Using a range of temperatures $T \in [250, 350]$ K guarantees an optimal sampling of the configuration space during the MD,

allowing for a more extended determination of the 2D-PES model. In the main text of the paper, we used the function plotted in Supplementary Fig. **15** to carry out an anharmonic vibrational analysis of the shuttling mode. The range of temperatures at which the model potential $V_{2D}(d_{O_1O_2}, \delta)$ has been derived is consistent with the temperatures where the PT shows a "sweet spot", supporting the outcome of our analysis.

## Supplementary Note VI. SPECIES POPULATION ANALYSIS

We present here the species population analysis carried out for the quantum simulations as a function of temperature. It is plotted in Supplementary Fig. **16**. One can see that

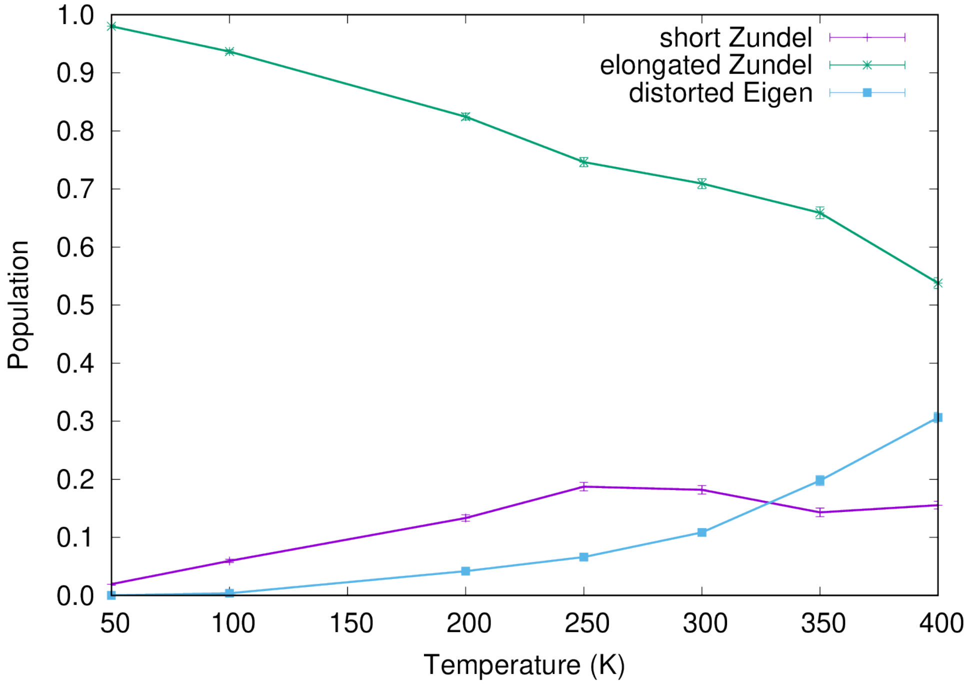


**Supplementary Figure 16**. Population of the short Zundel, elongated Zundel and distorted Eigen species, as evaluated from the QMC-driven PIMD trajectories, and plotted as a function of temperature. The species are defined based on their $d_{O_1O_2}$ distance computed for the centroids (see main text).

the short-Zundel population has a peak in the [250-300] K range, in accordance with the instanton analysis. Notice however that the population here is taken all over the sample, and not only over the instanton instances. As discussed in the main text, raising the temperature above the sweet spot region promotes a larger distorted-Eigen population. This is

detrimental for the short-Zundel population, which indeed falls down. The maximum in the short-Zundel population corresponds to the sweet spot in the PT, showing once again the key role played by the short-Zundel species in optimizing the PT.

It is interesting to study the impact of NQEs on the species population at 300 K. This is reported in Supplementary Fig. **17**. As discussed in the main text, at this temperature quantum effects favour the occurrence of the short-Zundel species with respect to the distorted Eigen states, penalised by a larger zero point energy, absent in classical calculations where the relative occurrence between the two species is reversed. This behaviour is in agreement with the outcome of Ref. [11], where a similar difference between classical and quantum populations has been found.

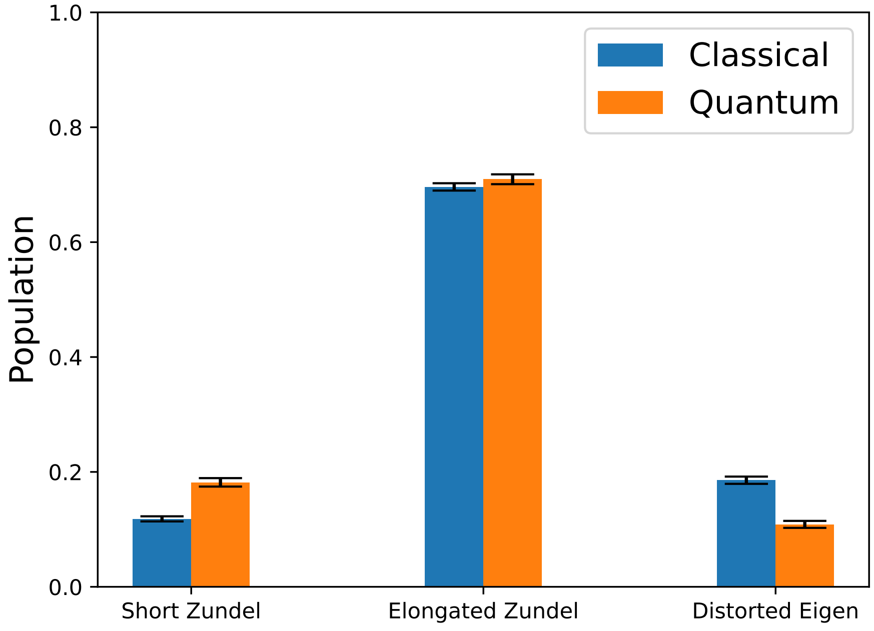


**Supplementary Figure 17**. Population of the short Zundel, elongated Zundel and distorted Eigen species at 300 K, evaluated from both classical and quantum QMC-driven MD.

## Supplementary Note VII. PROTON TRANSFER: ADIABATIC EVENTS VERSUS QUANTUM TUNNELING

The picture unveiled in this work proves that the synergy of NQEs and thermal effects is fundamental to understand PT in an aqueous environment. Beside the ZPE, NQEs can contribute to the proton diffusion by means of instantaneous tunneling, which can further accelerate the PT dynamics. Both adiabatic crossing boosted by the ZPE, and instantaneous tunneling are plausible and non-competing scenarios in our system. As we have seen, the former is certainly favoured by the shortness of $d_{O_1O_2}$ in the short-Zundel configurations, where the barrier is absent along the shuttling trajectory $\delta$, while the latter could sustain PT events even in case of high barriers, such as in Eigen-like configurations.

In order to assess the role played by quantum tunneling, we evaluate the localisation level of the excess proton during its dynamics, by computing the root-mean-square (RMS) displacement correlation functions[12], $\mathcal{R}_{d_{OH^+}}(\tau) = \langle |d_{OH^+}(0) - d_{OH^+}(\tau)|^2 \rangle$, in imaginary time $\tau \in [0, \beta\hbar]$, with $\beta = 1/k_B T$ and $d_{OH^+} = |\mathbf{q}_O - \mathbf{q}_{H^+}|$. For localised states, trapped in potential energy minima, $\mathcal{R}_{d_{OH^+}}(\tau)$ is flat in the intermediate $\tau$ range, while, for delocalised states, $\mathcal{R}_{d_{OH^+}}(\tau)$ is roughly parabolic [12]. A quantum particles undergoing a tunneling event will look like a free particle with parabolic behavior, as unaffected by the tunneled potential energy barrier.

The evolution of the RMS displacement correlations functions $\mathcal{R}_{d_{OH^+}}(\tau)$ with the temperature is depicted in Supplementary Fig. **18** for the instanton configurations, the most relevant for the PT description. Also in this case, we distinguish instantons belonging to the three different regimes. A striking difference is observed among the three temperatures investigated, i.e. 100 K, 250 K and 350 K. For the lowest temperature (Supplementary Fig. **18**(a)), the TS correlation function $\mathcal{R}_{d_{OH^+,TS}}(\tau)$ is flat, *i.e.* more localised, in all regimes. At intermediate temperatures (Supplementary Fig. **18**(b)), within the "sweet spot" range, the $\mathcal{R}_{d_{OH^+,traj}}(\tau)$ measured in the distorted-Eigen configurations is the closest to the free particle behavior, while the short-Zundel averages are clearly bound by the confining symmetric potential. This suggests that some PT events in the distorted Eigen, which need to overcome taller barriers, could be enhanced by quantum tunneling, as additional PT channel, beside the very effective short-Zundel TS mechanism. At the highest temperature (Supplementary Fig. **18**(c), all $\mathcal{R}_{d_{OH^+,traj}}(\tau)$ are very close to the free particle. However, in

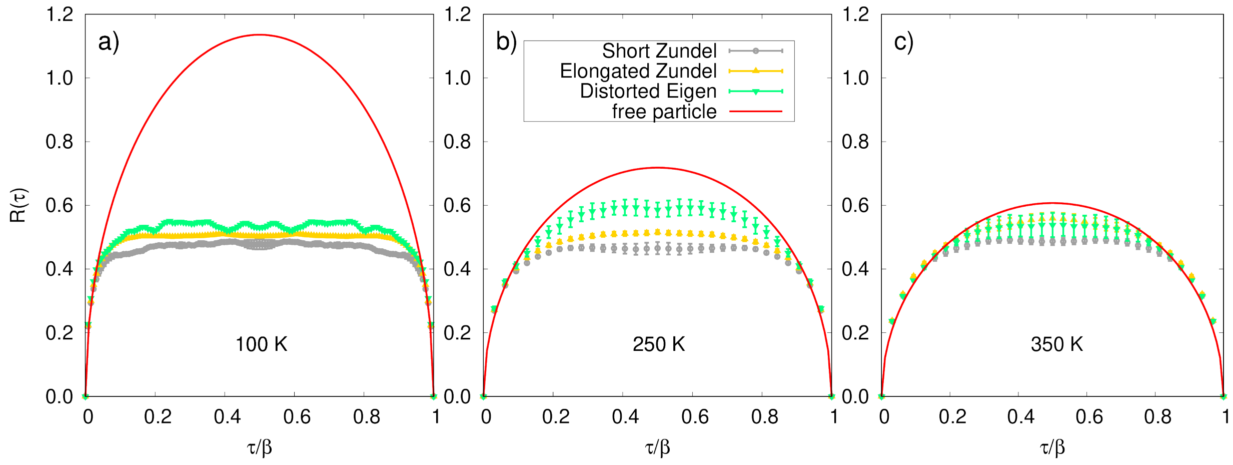


**Supplementary Figure 18**. Root mean square displacement $R(\tau)$ in quantum imaginary time $\tau^* \in [0, \beta\hbar/M] \equiv [0, 1]$ for the proton displacement with respect to the side oxygen atoms, $d_{OH^+} = |\mathbf{q}_O - \mathbf{q}_{H^+}|$, in the core of the $H_{13}O_6^+$ cation. Calculations are performed at 100 K, 250 K and 350 K, reported in panels a), b) and c), respectively. Samples are taken from the instanton population, and averages are performed in a species-selected way: short-Zundel (gray points), elongated-Zundel (yellow points) and Eigen-like species (green points) are separated, according to the $d_{O_1O_2}$ distance at which they occur. As reference, the root-mean-square displacement of the free proton at the given temperature is also reported in red solid lines.

this case, thermal effects, rather than quantum tunneling, make the instantons behave like free particles.

## SUPPLEMENTARY REFERENCES


[1] Casula, M., Attaccalite, C. & Sorella, S. Correlated geminal wave function for molecules: An efficient resonating valence bond approach. *J. Chem. Phys.* **121**, 7110 (2004).

[2] Sorella, S., Devaux, N., Dagrada, M., Mazzola, G. & Casula, M. Geminal embedding scheme for optimal atomic basis set construction in correlated calculations. *J. Chem. Phys.* **143**, 244112 (2015).

[3] Dagrada, M., Casula, M., Saitta, A. M., Sorella, S. & Mauri, F. Quantum Monte Carlo study of the protonated water dimer. *J. Chem. Theory Comput.* **10**, 1980–1993 (2014).

[4] Mouhat, F., Sorella, S., Vuilleumier, R., Saitta, A. M. & Casula, M. Fully quantum description of the zundel ion: Combining variational quantum Monte Carlo with path integral langevin dynamics. *J. Chem. Theory Comput.* **13**, 2400–2417 (2017).

[5] Neuscamman, E. Size consistency error in the antisymmetric geminal power wave function can be completely removed. *Phys. Rev. Lett.* **109**, 203001 (2012). URL `http://link.aps.org/doi/10.1103/PhysRevLett.109.203001`.

[6] Lee, K., Murray, E. D., Kong, L., Lundqvist, B. I. & Langreth, D. C. Higher-accuracy van der Waals density functional. *Phys. Rev. B* **82** (2010).

[7] Yu, Q. *et al.* q-AQUA: A many-body CCSD(T) water potential, including four-body interactions, demonstrates the quantum nature of water from clusters to the liquid phase. *The Journal of Physical Chemistry Letters* **13**, 5068–5074 (2022).

[8] Huang, X., Braams, B. J. & Bowman, J. M. Ab initio potential energy and dipole moment surfaces for $H_5O_2^+$. *J. Chem. Phys.* **122**, 044308 (2005). URL `http://scitation.aip.org/content/aip/journal/jcp/122/4/10.1063/1.1834500`.

[9] Casula, M., Filippi, C. & Sorella, S. Diffusion Monte Carlo method with lattice regularization. *Phys. Rev. Lett.* **95**, 1431 (2005).

[10] Decka, D., Schwaab, G. & Havenith, M. A THz/FTIR fingerprint of the solvated proton: evidence for Eigen structure and Zundel dynamics. *Phys. Chem. Chem. Phys.* **17**, 11898–11907 (2015).

[11] Calio, P. B., Li, C. & Voth, G. A. Resolving the structural debate for the hydrated excess proton in water. *Journal of the American Chemical Society* **143**, 18672–18683 (2021).

[12] Chandler, D. & Leung, K. Excess electrons in liquids: Geometrical perspectives. *Annu. Rev.*

*Phys. Chem.* **45**, 557–591 (1994).
